# Comparative effectiveness of nipple-sparing mastectomy and breast-conserving surgery on long-term prognosis in breast cancer

**DOI:** 10.3389/fendo.2023.1222651

**Published:** 2023-11-20

**Authors:** Qitong Chen, Limeng Qu, Yeqing He, Yueqiong Deng, Qin Zhou, Wenjun Yi

**Affiliations:** ^1^ Department of General Surgery, The Second Xiangya Hospital of Central South University, Changsha, Hunan, China; ^2^ Clinical Research Center for Breast Disease in Hunan Province, Changsha, Hunan, China

**Keywords:** breast cancer, nipple-sparing mastectomy (NSM), propensity score matching (PSM), Surveillance Epidemiology and End Results (SEER), breast-conserving surgery (BCS)

## Abstract

**Background:**

The frequency of nipple-sparing mastectomy (NSM) surgery is presently increasing. Nonetheless, there is a paucity of long-term prognosis data on NSM. This study compared the long-standing prognosis of NSM in relation to breast-conserving surgery (BCS).

**Methods:**

Population-level data for 438,588 female breast cancer patients treated with NSM or BCS and postoperative radiation from 2000 to 2018 were identified in the Surveillance, Epidemiology, and End Results (SEER) database; 321 patients from the Second Xiangya Hospital of Central South University were also included. Propensity score matching (PSM) was performed to reduce the influence of selection bias and confounding variables to make valid comparisons. The Kaplan–Meier analysis, log-rank test, and Cox regression were applied to analyze the data.

**Results:**

There were no significant differences in long-term survival rates between patients who underwent NSM and those who underwent BCS+radiotherapy (BCS+RT), as indicated by the lack of significant differences in overall survival (OS) (p = 0.566) and breast cancer-specific survival (BCSS) (p = 0.431). Cox regression indicated that NSM and BCS+RT had comparable prognostic values (p = 0.286) after adjusting for other clinicopathological characteristics. For OS and BCSS, subgroup analysis showed that the majority of patients achieved an analogous prognosis whether they underwent NSM or BCS. The groups had comparable recurrence-free survival (RFS), with no significant difference found (p = 0.873).

**Conclusions:**

This study offers valuable insights into the long-term safety and comparative effectiveness of NSM and BCS in the treatment of breast cancer. These findings can assist clinicians in making informed decisions on a case-by-case basis.

## Introduction

Breast cancer is a common disease and a leading cause of cancer-related death among women worldwide (1, 2). The primary approach in the comprehensive treatment of breast cancer is surgical intervention to remove the tumor. In the last century, there has been an evolution in the surgical approach for breast cancer from expanded radical surgery to modified radical mastectomy (MRM), with breast-conserving surgery (BCS) as the preferred surgical treatment approach (3). The concept of surgical treatment has changed from “maximum tolerable treatment” to “minimum effective treatment”. For patients with early-stage breast cancer, BCS combined with postoperative radiation has been widely used in clinical practice. This approach offers a less invasive option, less scarring, and a faster recovery time for patients. However, BCS may not be suitable for all patients, especially those with large tumors, multifocal tumors, or tumors that are located in a challenging position, recurrence after breast-conserving surgery, and small breast size.

In these cases, nipple-sparing mastectomy (NSM) involves removing the breast tissue completely while preserving the nipple-areola complex (NAC), and facilitating postoperative breast reconstruction becomes an alternative option (4). NSM has gained popularity due to its better aesthetic outcomes, reduced psychological impact, and improved quality of life for patients. Recent studies have suggested that NSM may offer some advantages over breast-conserving surgery, such as better cosmetic outcomes and a reduced risk of local recurrence (5, 6). However, NSM is a more complex and technically demanding procedure that requires specialized training and expertise. Furthermore, there is concern that preserving the nipple may increase the risk of cancer recurrence.

The safety of NSM has been confirmed. Shimo et al. reviewed data for 425 patients who underwent NSM for breast cancer, with a median follow-up of 46.8 months. The postoperative local recurrence rate was 5.8% (25/425), which was not statistically significant (p > 0.05) compared to the local recurrence rate of 5.6% (49/878) with conventional radical mastectomy during the same period. Moreover, the local recurrence rate after NSM surgery was only 2.3% (7). In a meta-analysis (8) of 5,594 breast cancer patients in 20 studies, Cruz et al. compared the overall survival, disease-free survival, and rate of local recurrence after NSM, skin-sparing mastectomy (SSM), and MRM in three surgical procedures. The differences in the overall survival rate, disease-free survival rate, and local recurrence rate of the three procedures were not statistically significant, and the recurrence rate in the nipple-areola after NSM was only 2.1%, which was not statistically significant compared with that of other surgical procedures (8).

Few studies with large sample sizes have investigated long-term outcomes following NSM, and the choice of NSM or BCS for better oncologic outcomes in T0–T2 breast cancer patients remains inconclusive. In our study, we sought to conduct a retrospective analysis and evaluation of long-term outcomes associated with different types of mastectomies, namely, NSM and BCS, by employing data sourced from the esteemed Surveillance, Epidemiology, and End Results (SEER) database as well as the Second Xiangya Hospital of Central South University. The optimal surgical approach should be tailored to each patient’s individual needs and circumstances. Our primary objective was to provide guidance for patients and clinicians by offering them additional evidence such that they could make informed decisions and select the most appropriate surgical option.

## Methods

### Data source

The collection of cancer incidence data of SEER (http://www.seer.cancer.gov) was accomplished through population-based cancer registries, covering a significant proportion of the U.S. population at approximately 47.9% (9). Patient-level information on all types of cancer from 21 cancer registries located throughout the United States is included in the SEER database. We extracted population-level data using the National Cancer Institute’s SEER cancer database and the Surveillance Research Program’s SEER*Stat software (version 8.4.0.1) available at www.seer.cancer.gov/seerstat. The registry included individuals who were diagnosed with cancer, and information was collected by the SEER registry regarding demographic data, tumor clinicopathological characteristics, treatment mode, and survival status of each individual, including the cause of death of the patient during follow-up.

Breast cancer patients who received BCS and NSM between 2014 and 2021 at the Second Xiangya Hospital of Central South University were considered for inclusion. This retrospective study was reviewed and approved by the Ethics Committee of the Second Xiangya Hospital of Central South University. The study adhered to the Strengthening the Reporting of Observational Studies in Epidemiology (STROBE) guidelines (10) for reporting.

### Patient selection

We defined cancers using the International Classification of Diseases for Oncology, Version 3 (ICD-O-3) and originally identified breast cancer cases. The coding rules for data collection are specified in the coding and staging manual of the SEER Program (11). Participants diagnosed with pathologically confirmed breast cancer from 1 January, 2000, to 31 December, 2018, were selected based on the following criteria: 1) female; 2) Tis, T1, and T2 stage disease; and 3) NSM (SEER surgery code 30) or BCS (codes 20–24) treatment. We adhered to the following exclusion criteria: 1) not a primary tumor, 2) incomplete follow-up data, 3) not American Joint Committee on Cancer (AJCC) M0 stage disease, and (4) unknown or indefinite N stage.

**Figure 1:**
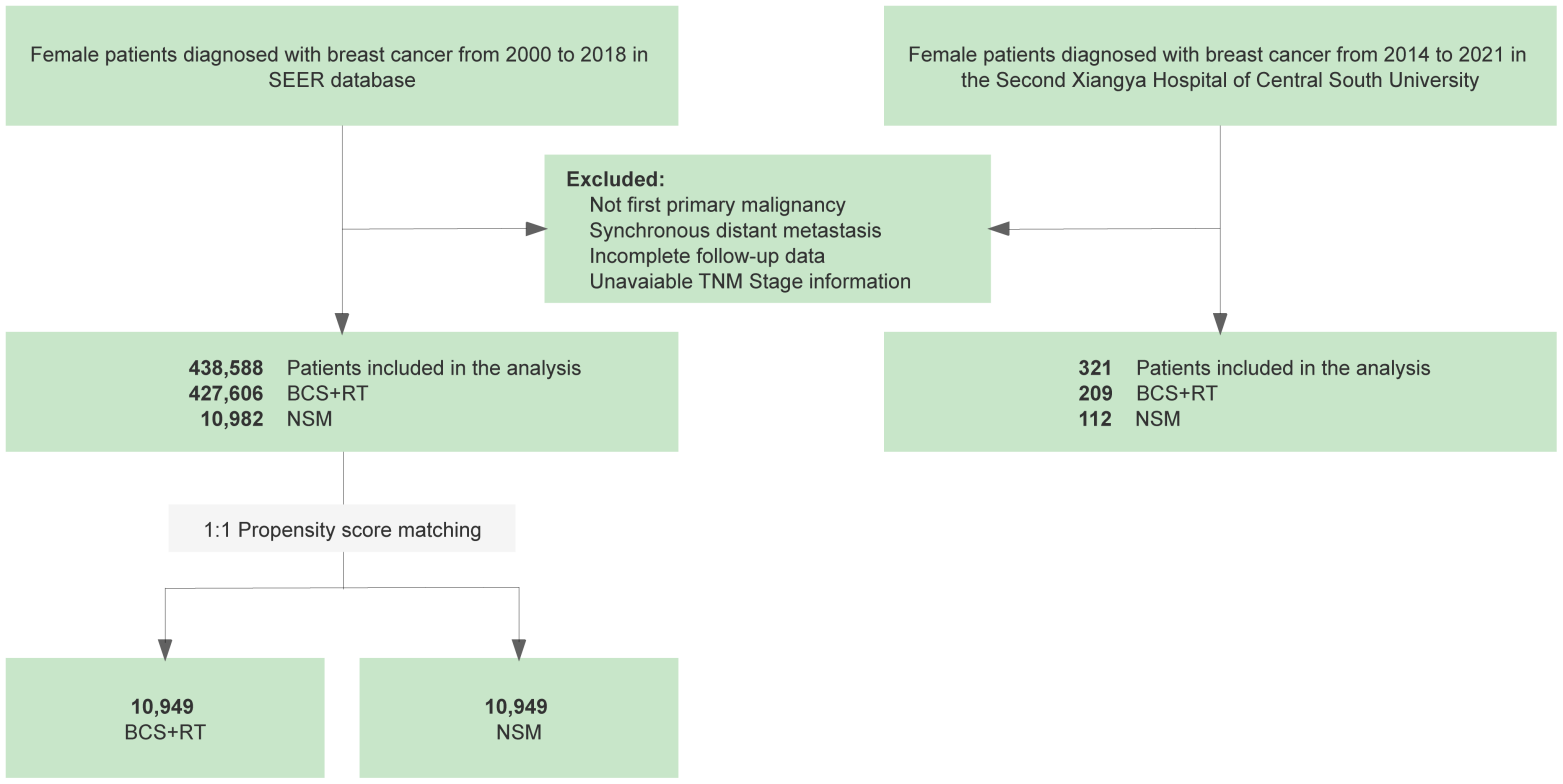
Flowchart of the retrospective study based on data from the Surveillance, Epidemiology, and End Results (SEER) database and the Second Xiangya Hospital of Central South University comparing nipple-sparing mastectomy (NSM) and breast-conserving surgery (BCS) for breast cancer treatment.

Similarly, a retrospective collection of data on breast cancer patients who had undergone BCS and NSM between 2014 and 2021 was carried out at the Second Xiangya Hospital of Central South University. Patients who were diagnosed with metastatic disease or had incomplete follow-up and clinicopathological data were excluded from this study. In the subsequent stage, patient demographics, tumor characteristics, and treatment data from the initial cancer diagnosis were meticulously recorded. Follow-up data were collected, which included the last visit in the system regarding the development of recurrence status. Finally, 438,588 and 321 patients were included in the SEER cohort and Xiangya cohort, respectively, for further analysis. Details regarding the selection procedure can be found in **Figure 1**.

We included the following demographic variables, clinicopathological characteristics, and treatment information of breast cancer patients from the SEER database before and after propensity score matching (PSM) (**Table 1**): age at diagnosis, year of diagnosis, marital status, race, histology, grade, breast-adjusted T stage, N stage, estrogen receptor (ER) status, progesterone receptor (PR) status, human epidermal growth factor receptor 2 (HER2) status, molecular subtype, surgery of primary site, and chemotherapy status. HER2 status data were not documented until January 2010, resulting in some patients with an unavailable HER2 status being enrolled in the present study (12). We transformed continuous variables and age at diagnosis into categorical variables using the following groups: ≤45, 46–65, and >65 years. We performed analyses of survival (months), vital status, and cause-specific death classification data to evaluate prognostic outcomes. We grouped patients according to the type of mastectomy they received: NSM or BCS with postoperative radiation.

**Table 1:** Clinicopathological characteristics of patients who underwent NSM or BCS for breast cancer in the SEER cohort.

Characteristics, n (%)	Before PSM				After PSM			
	Overall	BCS+RT	NSM	p-Value	Overall	BCS+RT	NSM	p-Value
	n = 438,588	n = 427,606	n = 10,982		n = 21,898	n = 10,949	n = 10,949	
Age								
≤45	51,626 (11.8)	47,826 (11.2)	3,800 (34.6)	<0.001	7,531 (34.4)	3,764 (34.4)	3,767 (34.4)	0.999
46–65	242,929 (55.4)	236,710 (55.4)	6,219 (56.6)		12,442 (56.8)	6,223 (56.8)	6,219 (56.8)	
>65	144,033 (32.8)	143,070 (33.5)	963 (8.8)		1,925 (8.8)	962 (8.8)	963 (8.8)	
Year of diagnosis								
2000–2004	98,470 (22.5)	98,255 (23.0)	215 (2.0)	<0.001	427 (1.9)	212 (1.9)	215 (2.0)	0.999
2005–2009	107,762 (24.6)	107,446 (25.1)	316 (2.9)		631 (2.9)	315 (2.9)	316 (2.9)	
2010–2013	93,175 (21.2)	91,305 (21.4)	1,870 (17.0)		3,740 (17.1)	1,870 (17.1)	1,870 (17.1)	
2014–2018	139,181 (31.7)	130,600 (30.5)	8,581 (78.1)		17,100 (78.1)	8,552 (78.1)	8,548 (78.1)	
Marital status								
Married	266,766 (60.8)	259,407 (60.7)	7,359 (67.0)	<0.001	14,709 (67.2)	7,380 (67.4)	7,329 (66.9)	0.745
Single	55,385 (12.6)	53,763 (12.6)	1,622 (14.8)		3,214 (14.7)	1,593 (14.5)	1,621 (14.8)	
DSW	100,415 (22.9)	98,881 (23.1)	1,534 (14.0)		3,072 (14.0)	1,538 (14.0)	1,534 (14.0)	
Unknown	16,022 (3.7)	15,555 (3.6)	467 (4.3)		903 (4.1)	438 (4.0)	465 (4.2)	
Race								
White	355,485 (81.1)	347,004 (81.2)	8,481 (77.2)	<0.001	16,950 (77.4)	8,498 (77.6)	8,452 (77.2)	0.822
Black	42,291 (9.6)	41,404 (9.7)	887 (8.1)		1,757 (8.0)	870 (7.9)	887 (8.1)	
Other	38,917 (8.9)	37,390 (8.7)	1,527 (13.9)		3,026 (13.8)	1,503 (13.7)	1,523 (13.9)	
Unknown	1,895 (0.4)	1,808 (0.4)	87 (0.8)		165 (0.8)	78 (0.7)	87 (0.8)	
Histology								
Ductal carcinoma	270,436 (61.7)	264,004 (61.7)	6,432 (58.6)	<0.001	12,878 (58.8)	6,447 (58.9)	6,431 (58.7)	0.961
Lobular carcinoma	46,375 (10.6)	45,077 (10.5)	1,298 (11.8)		2,589 (11.8)	1,296 (11.8)	1,293 (11.8)	
Other	121,777 (27.8)	118,525 (27.7)	3,252 (29.6)		6,431 (29.4)	3,206 (29.3)	3,225 (29.5)	
Grade								
I	102,562 (23.4)	100,713 (23.6)	1,849 (16.8)	<0.001	3,715 (17.0)	1,866 (17.0)	1,849 (16.9)	0.926
II	179,198 (40.9)	174,857 (40.9)	4,341 (39.5)		8,715 (39.8)	4,374 (39.9)	4,341 (39.6)	
III–IV	126,992 (29.0)	123,489 (28.9)	3,503 (31.9)		6,963 (31.8)	3,463 (31.6)	3,500 (32.0)	
Unknown	29,836 (6.8)	28,547 (6.7)	1,289 (11.7)		2,505 (11.4)	1,246 (11.4)	1,259 (11.5)	
T stage								
T1	267,981 (61.1)	262,729 (61.5)	5,252 (47.8)	<0.001	10,488 (47.9)	5,236 (47.8)	5,252 (48.0)	0.921
T2	83,592 (19.1)	80,501 (18.8)	3,091 (28.2)		6,191 (28.3)	3,109 (28.4)	3,082 (28.1)	
Tis	87,015 (19.8)	84,376 (19.7)	2,639 (24.0)		5,219 (23.8)	2,604 (23.8)	2,615 (23.9)	
N stage								
N0	366,538 (83.6)	357,850 (83.7)	8,688 (79.1)	<0.001	17,371 (79.3)	8,713 (79.6)	8,658 (79.1)	0.409
N1	60,342 (13.8)	58,421 (13.7)	1,921 (17.5)		3,829 (17.5)	1,908 (17.4)	1,921 (17.5)	
N2	8,473 (1.9)	8,205 (1.9)	268 (2.4)		506 (2.3)	240 (2.2)	266 (2.4)	
N3	3,235 (0.7)	3,130 (0.7)	105 (1.0)		192 (0.9)	88 (0.8)	104 (0.9)	
ER								
Positive	348,073 (79.4)	339,232 (79.3)	8,841 (80.5)	<0.001	17,659 (80.6)	8,823 (80.6)	8,836 (80.7)	0.971
Negative	61,234 (14.0)	59,504 (13.9)	1,730 (15.8)		3,471 (15.9)	1,742 (15.9)	1,729 (15.8)	
Unknown	29,281 (6.7)	28,870 (6.8)	411 (3.7)		768 (3.5)	384 (3.5)	384 (3.5)	
PR								
Positive	301,094 (68.7)	293,372 (68.6)	7,722 (70.3)	<0.001	15,426 (70.4)	7,709 (70.4)	7,717 (70.5)	0.938
Negative	100,109 (22.8)	97,470 (22.8)	2,639 (24.0)		5,293 (24.2)	2,655 (24.2)	2,638 (24.1)	
Unknown	37,385 (8.5)	36,764 (8.6)	621 (5.7)		1,179 (5.4)	585 (5.3)	594 (5.4)	
HER2								
Positive	22,573 (5.1)	21,111 (4.9)	1,462 (13.3)	<0.001	2,905 (13.3)	1,448 (13.2)	1,457 (13.3)	0.998
Negative	162,283 (37.0)	155,790 (36.4)	6,493 (59.1)		12,987 (59.3)	6,496 (59.3)	6,491 (59.3)	
Unknown	47,422 (10.8)	44,937 (10.5)	2,485 (22.6)		4,928 (22.5)	2,467 (22.5)	2,461 (22.5)	
Unavailable	206,310 (47.0)	205,768 (48.1)	542 (4.9)		1,078 (4.9)	538 (4.9)	540 (4.9)	
Molecular subtype								
HR+/HER2−	145,229 (33.1)	139,581 (32.6)	5,648 (51.4)	<0.001	11,285 (51.5)	5,639 (51.5)	5,646 (51.6)	0.984
HR+/HER2+	16,762 (3.8)	15,711 (3.7)	1,051 (9.6)		2,079 (9.5)	1,031 (9.4)	1,048 (9.6)	
HER2 enriched	5,763 (1.3)	5,357 (1.3)	406 (3.7)		819 (3.7)	414 (3.8)	405 (3.7)	
TNBC	16,901 (3.9)	16,065 (3.8)	836 (7.6)		1,687 (7.7)	851 (7.8)	836 (7.6)	
Unknown	253,933 (57.9)	250,892 (58.7)	3,041 (27.7)		6,028 (27.5)	3,014 (27.5)	3,014 (27.5)	
Chemotherapy								
Chemotherapy	127,856 (29.2)	123,807 (29.0)	4,049 (36.9)	<0.001	8,111 (37.0)	4,068 (37.2)	4,043 (36.9)	0.737
Chemotherapy-naïve/ unknown	310,732 (70.8)	303,799 (71.0)	6,933 (63.1)		13,787 (63.0)	6,881 (62.8)	6,906 (63.1)	

BCS, breast-conserving surgery; DSW, divorced/separated/widowed; ER, estrogen receptor; HER2, human epidermal growth receptor 2; HR, hormone receptor; NSM, nipple-sparing mastectomy; PSM, propensity score matching; PR, progesterone receptor; RT, radiotherapy; TNBC, triple-negative breast cancer; SEER, Surveillance, Epidemiology, and End Results.

### Endpoint

All patients who were enrolled in the study had complete follow-up data. Overall survival (OS) was the primary endpoint, which was defined as the duration between the date of diagnosis and the date of death from any cause. The secondary outcome measures included breast cancer-specific survival (BCSS) and recurrence-free survival (RFS). BCSS was defined as the duration between the initial diagnosis and death due to breast cancer. RFS was defined as the duration between the initial diagnosis and the occurrence of breast cancer recurrence or death. In the present study, OS and BCSS were analyzed in the SEER cohort, and RFS was analyzed in the Xiangya cohort.

### Statistical analysis

We conducted Pearson’s χ (2) test or Fisher’s exact test to evaluate the heterogeneity of categorical variables between the BCS and NSM groups. Categorical variables are reported as frequencies and percentages. To ensure that the baseline characteristics of patients were evenly distributed between the NSM and breast-conserving surgery+radiotherapy (BCS+RT) groups, we utilized PSM (13, 14) with the following parameters: 1:1 pairing and nearest-neighbor algorithms with a 0.05 caliper. Disparate demographic and clinicopathological features were adequately balanced after PSM, allowing for further analyses.

The Kaplan–Meier curves with the log-rank test for OS, BCSS, and RFS were constructed using the Kaplan–Meier method (15) via the R packages “survival” and “survminer” (16). Cox proportional hazards regression analysis was applied with the R packages “survival” and “survminer” (16) and was visualized using the R package “forestplot”. Statistical analyses and data visualization were performed using R (https://www.r-project.org/version4.2.2) and RStudio. All statistical tests were conducted as two-sided, and results were considered to be statistically significant if p < 0.05.

## Results

### Demographics and clinicopathological findings

After eliminating patients based on the exclusion criteria, 438,588 female breast cancer patients diagnosed between 2000 and 2018 were screened from the SEER database for further analysis. Among the candidates in the SEER cohort, 427,606 (97.5%) patients underwent BCS+RT; 10,982 (2.5%) patients underwent NSM. In patients undergoing NSM, the median patient age was 49.0 years (range: 19–97), 34.6% of the patients were ≤45 years old, and only 8.8% of the patients were >65 years old. In the BCS+RT group, the median patient age was 60.0 years (range: 18–100), 33.5% of the patients were >65 years old, and only 11.2% of the patients were ≤45 years old. Regarding the year of diagnosis in the NSM group, 2010–2013 and 2014–2018 were reported for the majority of patients (1,870, 17.0% and 8,581, 78.1%), but the year of diagnosis tended to be evenly distributed in the BCS+RT group. Compared to the NSM group (67.0%), the rate of marital status was lower in the BCS+RT group (60.7%). The majority of patients were white in both groups (81.2%, 77.2%). For patients in different surgery groups, the histologic type breast carcinoma had a similar rate. In the grading system, patients who received BCS+RT had an obviously higher tendency toward grade I than those who received NSM (23.6% *vs.* 16.8%). Regarding tumor stage, stages T1, T2, and Tis were found in the BCS+RT group (61.5%, 18.8%, and 19.7%, respectively) and the NSM group (47.8%, 28.2%, and 24.0%, respectively). The proportion of T2 tumor stages varied greatly. The NSM group had a higher rate (20.9%) of lymph node metastasis than the BCS+RT group (16.3%). For ER and PR status, the positive rates of BCS+RT (79.3%, 68.6%) were slightly lower than those of NSM (80.5%, 70.3%). Based on available HER2 data, a positive HER2 status was found in both the BCS+RT (11.9%, 21,111/155,790) and NSM (18.4%, 1,462/6,493) groups. Except for patients with unknown molecular subtype data, HR+/HER2− (BCS+RT: 79.0% *vs.* NSM: 71.1%), HR+/HER2+ (8.9% *vs.* 13.2%), HER2-enriched (3.0% *vs.* 5.1%), and TNBC (9.1% *vs.* 10.5%) types were documented. In total, 29.0% of patients in the BCS+RT group received chemotherapy, with 36.9% in the NSM group.

**Table 1** displays a comparison of clinicopathological characteristics between the BCS+RT and NSM groups. The two groups showed significant differences in most variables (p < 0.05). PSM was employed to minimize the potential impact of prognostic confounders on the accuracy of the results. The PSM cohort consisted of 21,898 subjects, with 10,949 patients in both the BCS+RT and NSM groups. There were no significant differences in any key methodological characteristics between the two groups. **Table 1** summarizes the baseline demographics, clinicopathological features, and therapy characteristics of the SEER cohort before and after PSM.

A total of 321 breast cancer patients who underwent NSM (n = 112, 34.9%) and BCS+RT (209, 65.1%) at the Second Xiangya Hospital of Central South University, Changsha, China, were included. The median age at the time of initial cancer diagnosis was 44 years, with a range of 21 to 86 years. Similar to the SEER cohort, the age of diagnosis in the NSM group (42, range 24–58) was younger than that in the BCS+RT group (46, range 21–86). Other characteristics of the Xiangya cohort had an analogous distribution in both groups. The baseline clinical, pathological, and other features of the Xiangya cohort are summarized in **Table 2**.

**Table 2:** Clinicopathological characteristics of patients who underwent NSM or BCS for breast cancer in the Xiangya cohort.

Characteristics	Overall	BCS	NSM	p-Value
	n = 321	n = 209	n = 112	
**Age (mean (SD))**	44.2 (9.9)	46.0 (10.6)	40.9 (7.6)	<0.001*
Menopausal status (%)				
Premenopausal	251 (78.2)	46 (69.9)	105 (93.8)	<0.001*
Postmenopausal	70 (21.8)	63 (30.1)	7 (6.2)	
Site (%)				
Left	166 (51.7)	112 (53.6)	54 (48.2)	0.423
Right	155 (48.3)	97 (46.4)	58 (51.8)	
Grade (%)				
1	56 (17.4)	30 (14.4)	26 (23.2)	0.136
2	205 (63.9)	138 (66.0)	67 (59.8)	
3	60 (18.7)	41 (19.6)	19 (17.0)	
T stage (%)				
T1	178 (55.5)	120 (57.4)	58 (51.8)	0.782
T2	120 (37.4)	75 (35.9)	45 (40.2)	
T3	6 (1.9)	4 (1.9)	2 (1.8)	
Tis	17 (5.3)	10 (4.8)	7 (6.2)	
N stage (%)				
N0	228 (71.0)	149 (71.3)	79 (70.5)	0.900
N1	55 (17.1)	37 (17.7)	18 (16.1)	
N2	24 (7.5)	15 (7.2)	9 (8.0)	
N3	14 (4.4)	8 (3.8)	6 (5.4)	
Hormone receptor status (%)				
Negative	79 (24.6)	48 (23.0)	31 (27.7)	0.425
Positive	242 (75.4)	161 (77.0)	81 (72.3)	
HER2 receptor status (%)				
Negative	243 (75.7)	161 (77.0)	82 (73.2)	0.533
Positive	78 (24.3)	48 (23.0)	30 (26.8)	
**Ki-67 index (mean (SD))**	31.9 (24.0)	32.3 (25.2)	31.2 (21.9)	0.705
ALND (%)				
Not performed	157 (48.9)	103 (49.3)	54 (48.2)	0.948
Performed	164 (51.1)	106 (50.7)	58 (51.8)	
SLNB (%)				
Not performed	150 (46.7)	95 (45.5)	55 (49.1)	0.612
Performed	171 (53.3)	114 (54.5)	57 (50.9)	
**Lymph node metastasis (mean (SD))**	1.3 (3.3)	1.2 (3.1)	1.5 (3.6)	0.479
**Lymph node examined (mean (SD))**	10.3 (7.2)	10.3 (7.2)	10.3 (7.2)	0.955

BCS, breast-conserving surgery; NSM, nipple-sparing mastectomy; HER2, human epidermal growth factor receptor 2; ALND, axillary lymph node dissection; SLN, sentinel lymph node biopsy.

* Statistically significant.

### Comparison of survival rates of different surgeries

**Figure 2:**
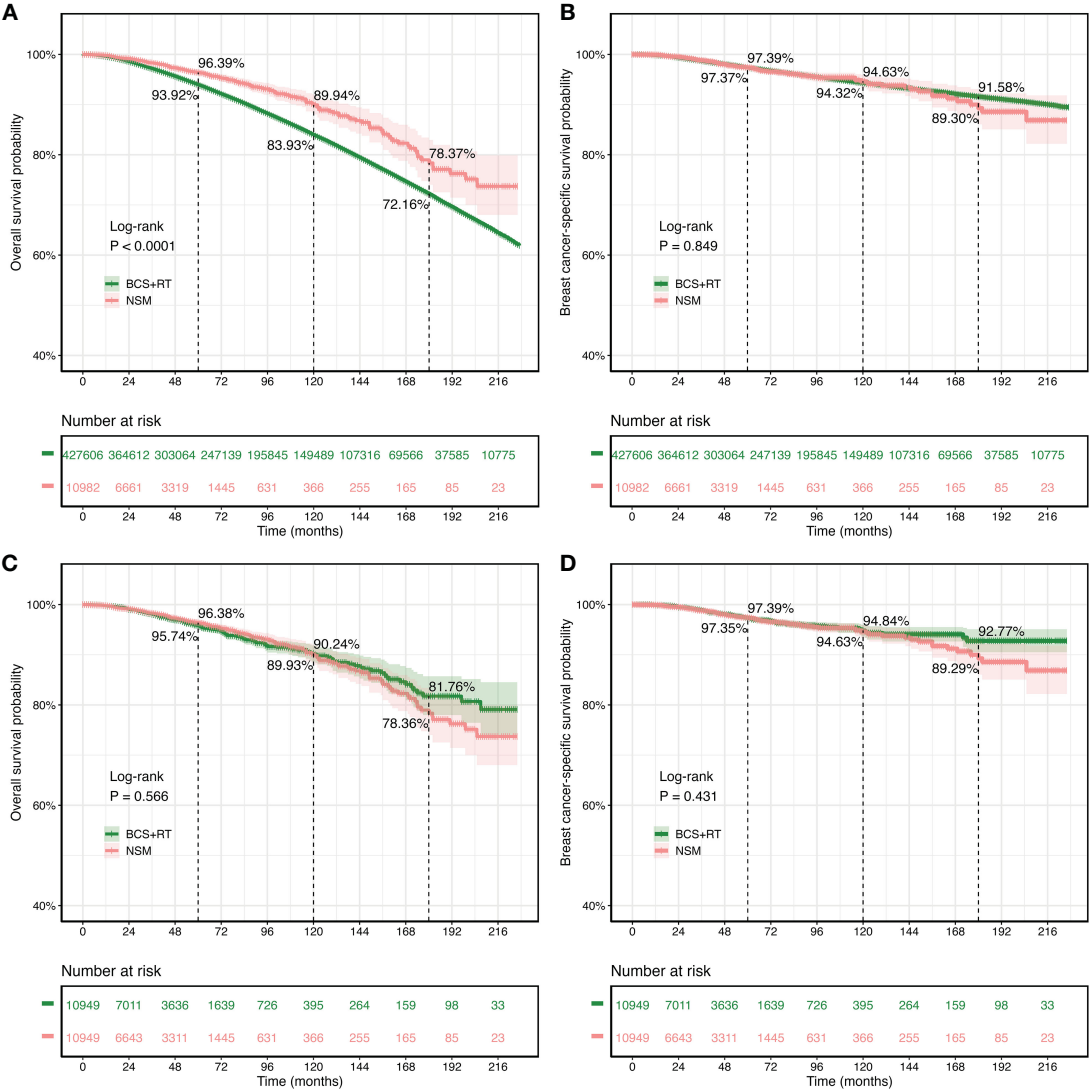
Kaplan–Meier survival curves for overall survival (OS) and breast cancer-specific survival (BCSS) in patients treated with nipple-sparing mastectomy (NSM) or breast-conserving surgery (BCS). **(A, B)** The original Surveillance, Epidemiology, and End Results (SEER) cohort. **(C, D)** The propensity score matching cohort.

The median follow-up for the original SEER dataset and the PSM SEER cohort was 85.0 and 33.0 months, respectively. **Figure 2A** shows that in the original SEER cohort, the NSM group had higher 5-year (96.39% *vs.* 93.92%), 10-year (89.94% *vs.* 83.93%), and 15-year (78.37% *vs.* 72.16%) OS rates than the BCS+RT group. In the NSM group, the cumulative 5-, 10-, and 15-year BCSS rates for the original SEER dataset were 97.39%, 94.63%, and 91.58%, respectively. In comparison, the cumulative 5-, 10-, and 15-year BCSS rates for the patients who underwent BCS+RT were 97.37%, 94.32%, and 89.30%, respectively. The BCSS rates of the NSM group were similar to those of the BCS+RT group, as shown in **Figure 2B**. The log-rank test and Kaplan–Meier survival curves revealed that the p-values for OS and BCSS between the NSM group and the BCS+RT group were p < 0.0001 and p = 0.849, respectively.

The survival analysis included 10,949 patients who received NSM and 10,949 patients who received BCS+RT after PSM matching. The results showed that the 5-year OS rates were comparable between the NSM and BCS+RT groups (96.38% *vs.* 95.74%). Additionally, the 10-year (90.24% *vs.* 89.93%) and 15-year (78.36% *vs.* 81.76%) OS rates in the NSM group were similar to those in the BCS+RT group, with no statistically significant difference (p = 0.566, **Figure 2C**). Similarly, the 5-year (97.39% *vs.* 97.35%), 10-year (94.63% *vs.* 94.84%), and 15-year (89.29% *vs.* 92.77%) BCSS rates were similar (p = 0.431, **Figure 2D**).

### Univariate and multivariate Cox analyses for OS and BCSS

Univariate and multivariate Cox regression analyses were performed for the SEER cohort after PSM to evaluate each prognostic factor (**Table 3** for OS and **Table 4** for BCSS). Age, year of diagnosis, marital status, race, histology, grade, T stage, N stage, molecular subtype, and chemotherapy were significant (p < 0.05) in univariate analysis for OS, while surgery was not (p = 0.566). Further multivariate regression analysis showed that age, year of diagnosis, marital status, race, grade, T stage, N stage, and molecular subtype were independent prognostic factors (p < 0.05) for OS (**Table 3**), while histology (p = 0.079), chemotherapy (p = 0.828), and surgery (p = 0.623) were not. For BCSS, the majority of characteristics showed significance (p < 0.05) in univariate analysis, except for the year of diagnosis (p = 0.244) and surgery (p = 0.432). Multivariate regression analysis revealed that year of diagnosis (p = 0.085), marital status (p = 0.086), chemotherapy (p = 0.252), and surgery (p = 0.286) may not be independent prognostic factors.

**Table 3:** Univariate and multivariate Cox analyses for OS in the SEER cohort after PSM.

Characteristic	Univariate Cox			Multivariate Cox		
	Hazard ratio	95% CI	p-Value	Hazard ratio	95% CI	p-Value
**Age**			<0.001			<0.001
≤45	1.00	—		1.00	—	
46–65	0.77	0.64, 0.94		0.89	0.73, 1.09	
>65	3.21	2.59, 3.97		3.39	2.67, 4.32	
**Year**			<0.001			<0.001
2000–2004	1.00	—		1.00	—	
2005–2009	0.72	0.52, 0.99		0.65	0.47, 0.90	
2010–2013	0.67	0.50, 0.89		0.61	0.40, 0.94	
2014–2018	0.48	0.35, 0.66		0.45	0.29, 0.70	
**Marital status**			<0.001			<0.001
Married	1.00	—		1.00	—	
Single	1.54	1.23, 1.93		1.41	1.12, 1.78	
DSW	2.29	1.89, 2.77		1.59	1.30, 1.95	
Unknown	1.64	1.10, 2.45		1.56	1.04, 2.33	
**Race**			<0.001			<0.001
White	1.00	—		1.00	—	
Black	2.06	1.67, 2.54		1.57	1.26, 1.95	
Other	0.56	0.40, 0.78		0.65	0.46, 0.90	
Unknown	not available	not available		not available	not available	
**Histology**			<0.001			0.079
Ductal carcinoma	1.00	—		1.00	—	
Lobular carcinoma	0.88	0.69, 1.11		1.05	0.81, 1.36	
Other	0.40	0.32, 0.49		0.70	0.49, 0.98	
**Grade**			<0.001			0.003
I	1.00	—		1.00	—	
II	1.22	0.93, 1.61		1.09	0.82, 1.45	
III–IV	2.03	1.56, 2.65		1.56	1.15, 2.10	
Unknown	1.21	0.83, 1.78		1.29	0.87, 1.93	
**T stage**			<0.001			<0.001
T1	1.00	—		1.00	—	
T2	2.67	2.24, 3.19		1.87	1.54, 2.26	
Tis	0.54	0.41, 0.69		0.89	0.57, 1.39	
**N stage**			<0.001			<0.001
N0	1.00	—		1.00	—	
N1	2.40	1.99, 2.90		1.70	1.38, 2.09	
N2	5.47	4.13, 7.24		3.38	2.46, 4.65	
N3	7.50	5.14, 11.0		4.18	2.77, 6.30	
**Molecular subtype**			<0.001			<0.001
HR+/HER2−	1.00	—		1.00	—	
HR+/HER2+	0.73	0.49, 1.08		0.65	0.43, 0.98	
HER2 enriched	1.24	0.77, 2.01		1.12	0.68, 1.83	
TNBC	3.67	2.91, 4.63		2.79	2.13, 3.65	
Unknown	0.85	0.69, 1.05		1.03	0.70, 1.52	
**Chemotherapy**			<0.001			0.828
Chemotherapy	1.00	—		1.00	—	
Chemotherapy-naïve/ unknown	0.41	0.35, 0.49		0.97	0.77, 1.23	
**Surgery**			0.566			0.623
BCS+RT	1.00	—		1.00	—	
NSM	0.95	0.81, 1.12		0.96	0.82, 1.13	

BCS, breast-conserving surgery; CI, confidence interval; DSW, divorced/separated/widowed; ER, estrogen receptor; HER2, human epidermal growth receptor 2; HR, hormone receptor; NSM, nipple-sparing mastectomy; PSM, propensity score matching; PR, progesterone receptor; RT, radiotherapy; TNBC, triple-negative breast cancer; SEER, Surveillance, Epidemiology, and End Results.— means Control group for Cox regression analysis.

**Table 4:** Univariate and multivariate Cox analyses for BCSS in the SEER cohort after PSM.

Characteristic	Univariate Cox			Multivariate Cox		
	Hazard ratio	95% CI	p-Value	Hazard ratio	95% CI	p-Value
**Age**			<0.001			<0.001
≤45	1.00	—		1.00	—	
46-65	0.53	0.42, 0.67		0.72	0.57, 0.91	
>65	1.02	0.72, 1.43		1.36	0.93, 1.99	
**Year**			0.244			0.085
2000–2004	1.00	—		1.00	—	
2005–2009	0.88	0.54, 1.44		0.75	0.46, 1.25	
2010–2013	0.95	0.61, 1.46		0.64	0.31, 1.33	
2014–2018	0.73	0.46, 1.15		0.48	0.23, 1.02	
**Marital status**			0.001			0.086
Married	1.00	—		1.00	—	
Single	1.54	1.16, 2.04		1.29	0.96, 1.73	
DSW	1.61	1.22, 2.11		1.39	1.05, 1.85	
Unknown	1.37	0.79, 2.35		1.27	0.74, 2.19	
**Race**			<0.001			0.001
White	1.00	—		1.00	—	
Black	2.11	1.60, 2.78		1.59	1.19, 2.13	
Other	0.72	0.49, 1.07		0.72	0.49, 1.07	
Unknown	not available	not available		not available	not available	
**Histology**			<0.001			<0.001
Ductal carcinoma	1.00	—		1.00	—	
Lobular carcinoma	0.83	0.62, 1.13		1.27	0.91, 1.77	
Other	0.13	0.08, 0.19		0.27	0.13, 0.55	
**Grade**			<0.001			<0.001
I	1.00	—		1.00	—	
II	1.96	1.22, 3.13		1.47	0.91, 2.38	
III–IV	4.89	3.12, 7.65		2.70	1.65, 4.42	
Unknown	1.62	0.84, 3.10		1.83	0.94, 3.56	
**T stage**			<0.001			<0.001
T1	1.00	—		1.00	—	
T2	4.18	3.31, 5.29		2.18	1.69, 2.81	
Tis	0.25	0.15, 0.42		1.26	0.50, 3.20	
**N stage**			<0.001			<0.001
N0	1.00	—		1.00	—	
N1	4.16	3.27, 5.31		2.16	1.66, 2.80	
N2	11.30	8.23, 15.6		5.64	3.93, 8.11	
N3	15.90	10.4, 24.1		5.94	3.76, 9.38	
**Molecular subtype**			<0.001			<0.001
HR+/HER2−	1.00	—		1.00	—	
HR+/HER2+	0.70	0.42, 1.16		0.52	0.31, 0.88	
HER2 enriched	1.75	1.04, 2.94		1.21	0.71, 2.07	
TNBC	4.79	3.66, 6.27		3.04	2.22, 4.14	
Unknown	0.58	0.43, 0.79		0.94	0.49, 1.80	
**Chemotherapy**			<0.001			0.252
Chemotherapy	1.00	—		1.00	—	
Chemotherapy-naïve/ unknown	0.17	0.14, 0.22		0.83	0.59, 1.15	
**Surgery**			0.432			0.286
BCS+RT	1.00	—		1.00	—	
NSM	1.09	0.88, 1.35		1.12	0.91, 1.39	

BCS, breast-conserving surgery; CI, confidence interval; DSW, divorced/separated/widowed; ER, estrogen receptor; HER2, human epidermal growth receptor 2; HR, hormone receptor; NSM, nipple-sparing mastectomy; PSM, propensity score matching; PR, progesterone receptor; RT, radiotherapy; TNBC, triple-negative breast cancer; SEER, Surveillance, Epidemiology, and End Results.— means Control group for Cox regression analysis.

### Subgroup analysis

**Figure 3:**
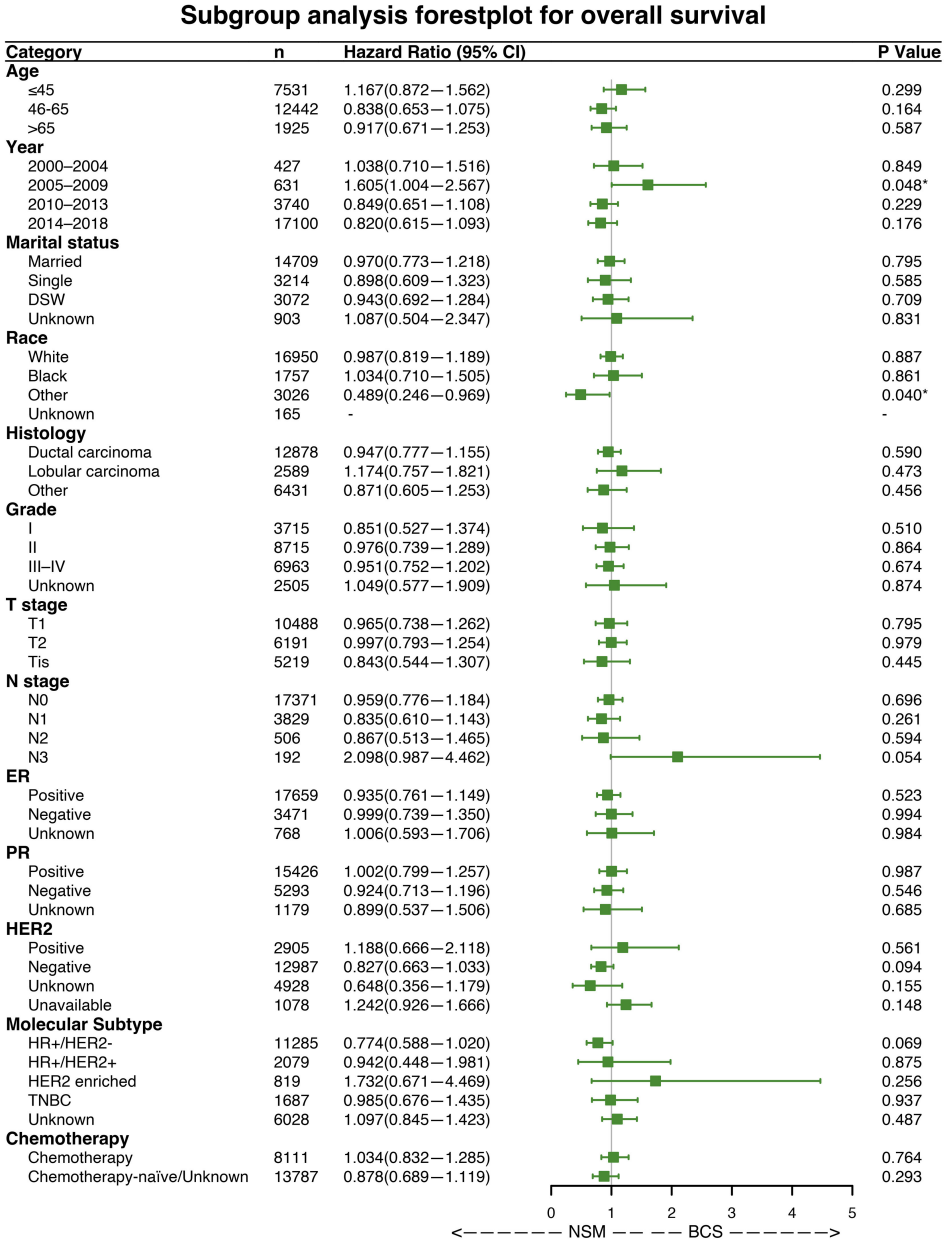
Subgroup analysis of overall survival (OS). Forest plot of univariate Cox analysis with hazard ratios and 95% confidence intervals. "-" means as “Control group for Cox regression analysis”. "*" means the p-value <0.05.

**Figure 4:**
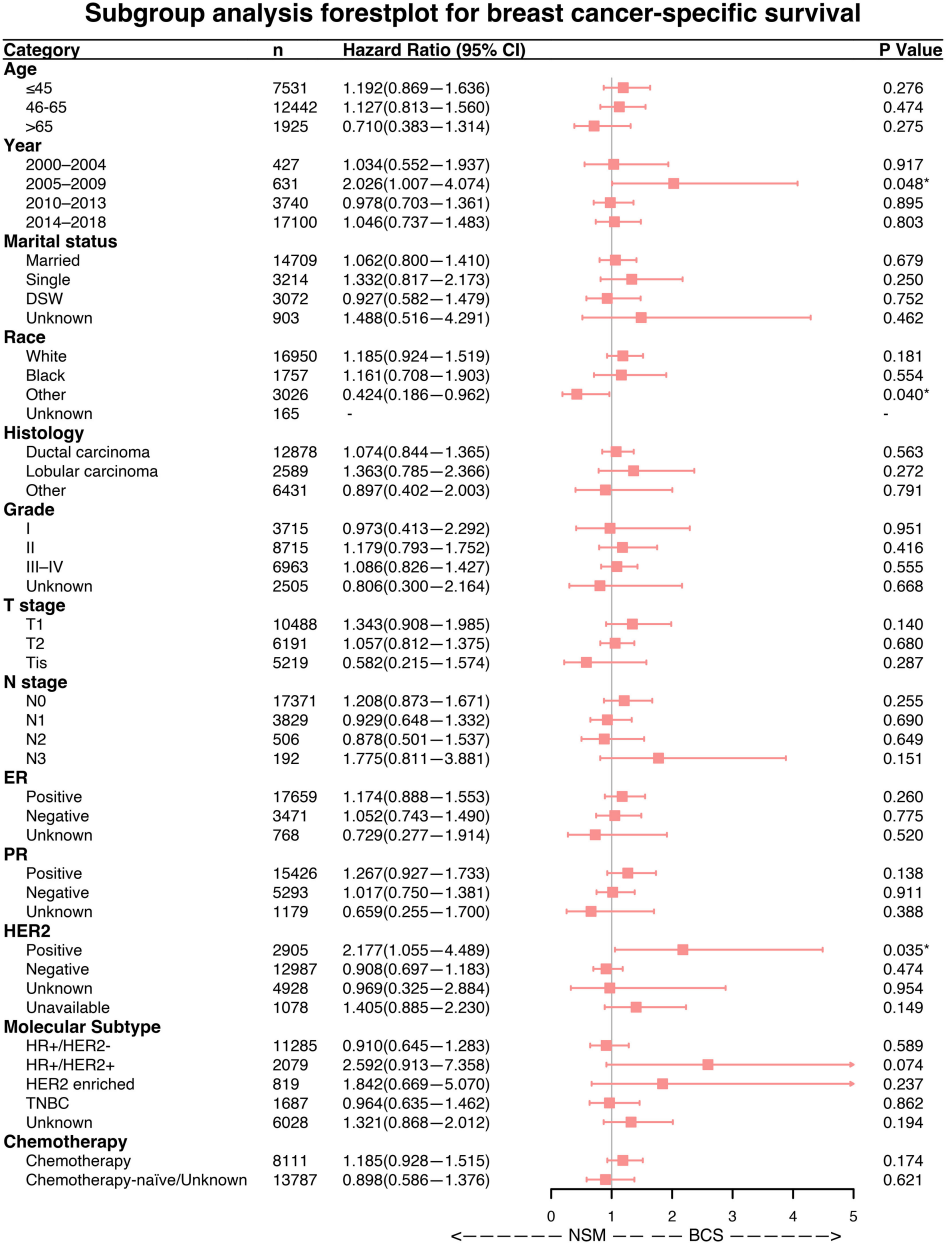
Subgroup analysis of breast cancer-specific survival (BCSS). Forest plot of univariate Cox analysis with hazard ratios and 95% confidence intervals. "-" means as “Control group for Cox regression analysis”. "*" means the p-value <0.05.

Forest plots indicated that NSM and BCS+RT had similar efficacy in terms of OS for most subgroups. However, NSM showed a greater advantage in the other race subgroup (HR = 0.489, p = 0.040) and a worse outcome in the subgroup diagnosed between 2005 and 2009 (HR = 1.605, p = 0.048), as illustrated in **Figure 3**. Regarding BCSS, significant differences were observed in a few variables, namely, the subgroup diagnosed between 2005 and 2009 (HR = 2.026, p = 0.048), the other race subgroup (HR = 0.424, p = 0.040), and the HER2-positive status subgroup (HR = 2.177, p = 0.035), as shown in **Figure 4**. These findings suggest that NSM may have comparable prognostic value to BCS+RT in breast cancer patients.

### Survival analysis for RFS and BRCA1/2 mutation

**Figure 5:**
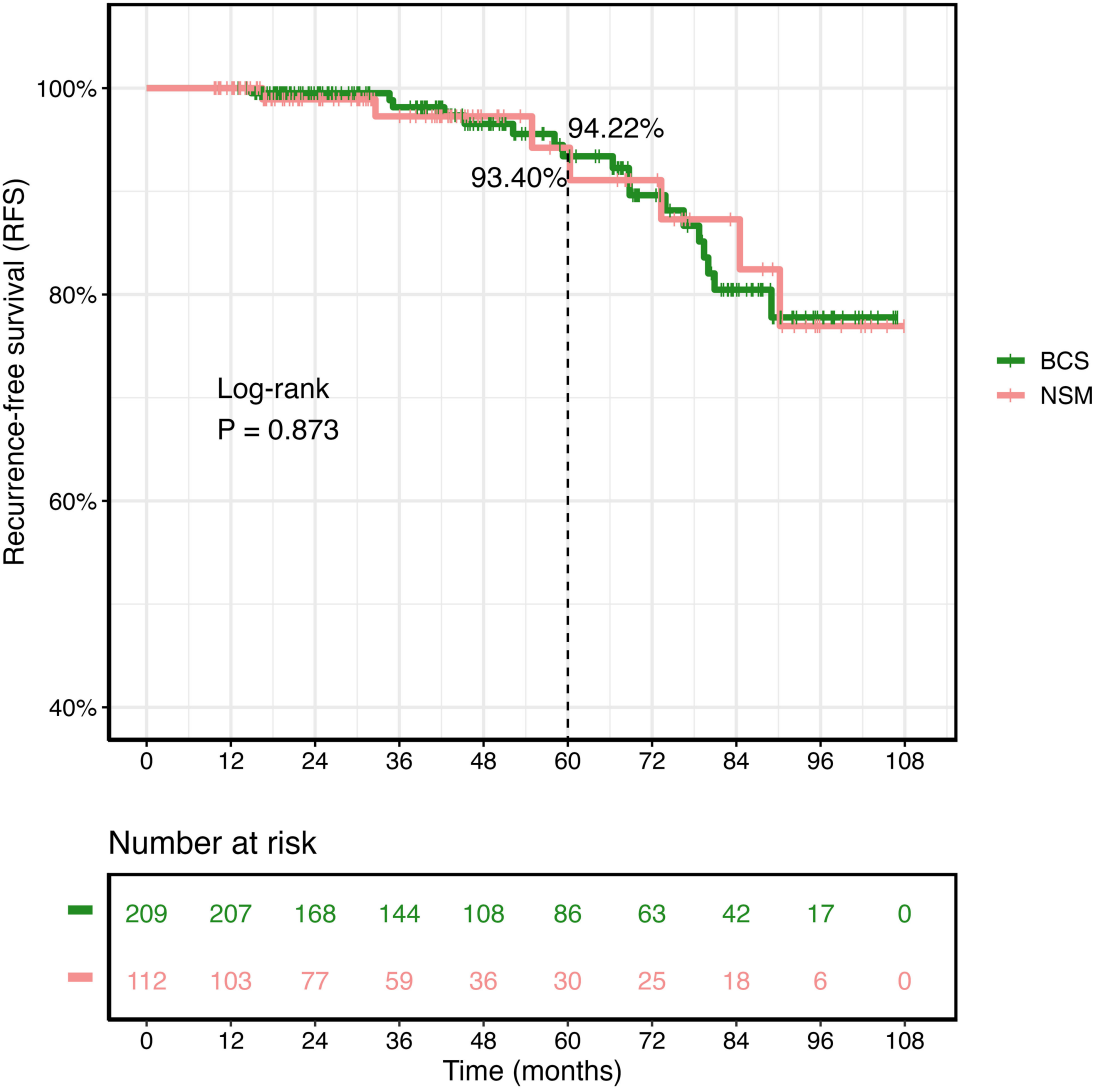
Kaplan–Meier survival curves of recurrence-free survival (RFS) in the Second Xiangya Hospital of Central South University cohort.

As of December 2022, the median follow-up for the 321 female patients in the Xiangya cohort was 45.0 months. The Kaplan–Meier survival curves and the log-rank test were used to compare recurrence rates between the NSM and BCS+RT groups, with no significant difference detected (p = 0.873, as shown in **Figure 5**). In this study, the BRCA1/2 germline mutation status was evaluated in 46 of 321 patients, of whom 12 were found to carry a positive BRCA1/2 mutation. The NSM surgery group harbored the majority of these mutations (n = 7, 36.8%, p = 0.293), as shown in **Table 5**.

**Table 5:** BRCA1/2 mutation of patients undergoing NSM and BCS for breast cancer in the Xiangya cohort.

BRCA1/2 mutation	Overall	BCS	NSM	p-Value
Positive	12 (26.1)	5 (18.5)	7 (36.8)	0.293
Negative	34 (73.9)	22 (81.5)	12 (63.2)	
Total	46	27	19	

BCS, breast-conserving surgery; NSM, nipple-sparing mastectomy.

## Discussion

In this study, which had a substantial sample size, we conducted an analysis of a cohort comprising 438,588 candidates in the SEER database and 21,898 patients in the PSM cohort. The study period spanned from 2000 to 2018. Our findings indicated that patients in the NSM group had similar OS and BCSS rates to those in the BCS+RT group. To our knowledge, this is the first and largest statistical study to directly assess the long-term efficacy of NSM in patients with T0–T2 stage and non-metastatic breast carcinoma based on a large population. Additionally, we employed RFS analyses of 321 individuals from the Second Xiangya Hospital of Central South University between 2014 and 2021. The recurrence rates between the NSM and BCS+RT groups were comparable.

Over the past century, there has been a continuous evolution in surgical techniques for breast cancer. The approach of simultaneously ensuring complete tumor removal and preserving breast aesthetics has emerged as the current trend in its development. In the 1960s, Freeman first proposed the concept of nipple-areola complex-sparing mastectomy (17). However, the safety of this procedure for the treatment of breast cancer has been debated. In recent years, the hesitation to provide NSM to cancer patients stems from concerns about the increased risk of local recurrence and the possibility of residual breast tissue leading to breast cancer in the future. Another study with a median follow-up of more than 5 years reported that the local recurrence rate was between 2% and 11.7%, with a recurrence rate following NAC between 1.3% and 3.7% (18–20). Current NSM approaches involve more complete removal of breast and nipple duct tissue, resulting in more comprehensive removal (21). NSM has gained increasing acceptance as a viable option for breast cancer treatment and risk reduction purposes (22).

This study found that both BCS and NSM had similar OS and BCSS outcomes. However, individuals who received NSM were typically younger. Moreover, NSM had a wider range of indications than BCS. Women who have mutations in BRCA1 and BRCA2 genes are at a high risk of developing breast cancer, with an estimated annual risk of 2%–3% and a lifetime risk of approximately 70% (23); furthermore, bilateral prophylactic mastectomies remain the most effective approach for reducing breast cancer risk (24). In young patients with high-risk factors, such as BRCA gene mutation carriers, NSM may be a better option due to its ability to provide better safety while maintaining breast shape integrity.

The present study had several limitations. First, the SEER database lacks information on various potential prognosis-related factors, such as details and regimens of radiotherapy, endocrine therapy, and targeted anti-HER2 therapies. Second, there may be variations in the diagnosis, therapeutic strategies, and follow-up of patients across institutions in different countries. Third, our study was retrospective in nature, and despite utilizing PSM and Cox regression statistical methods to reduce selection bias and increase the reliability of our results, we cannot entirely rule out the possibility of selection bias. Moreover, it is essential to note that “NSM” was entirely separate after 2010 but that part of “NSM” was included in total mastectomy before 2010 due to the SEER database’s surgery coding system (25). Finally, the sample size of the Chinese cohort was smaller than that of the SEER cohort, and our study lacked data on local recurrence. We aim to address these limitations in our future research.

## Conclusions

In conclusion, both NSM and BCS are viable surgical options for breast cancer treatment. However, their respective advantages and disadvantages must be carefully evaluated on a case-by-case basis. Our study shows that NSM is an oncologically safe and effective surgical treatment and that it can be recommended for female patients with early-stage breast cancer in the clinic. Nonetheless, randomized controlled clinical trials with long-term follow-up are needed to further evaluate the advantages of NSM for patients.

## Data availability statement

Publicly available datasets were analyzed in this study. This data can be found here: https://seer.cancer.gov/.

## Ethics statement

The studies involving humans were approved by the Ethics Committee of the Second Xiangya Hospital of Central South University. The studies were conducted in accordance with the local legislation and institutional requirements. The participants provided their written informed consent to participate in this study.

## Author contributions

Study design and idea construction: WY, QZ, and QC. Data collection and crosscheck: LQ, YH, YD, and QZ. Statistical analysis and data visualization: QC and QZ. Manuscript drafting and revision: QZ, LQ, YH, YD, QC, and WY. All authors contributed to the article and approved the submitted version.
